# Photomapping electrically coupled networks of the mature thalamus and cortex

**DOI:** 10.1126/sciadv.aed7447

**Published:** 2026-10-14

**Authors:** Mitchell J. Vaughn, David S. Uygun, Radhika Basheer, Kevin J. Bender, Julie S. Haas

**Affiliations:** ^1^ Department of Biological Sciences, Lehigh University, Bethlehem, PA 18015, USA.; ^2^ VA Boston Healthcare System; and Department of Psychiatry, Harvard Medical School, West Roxbury, MA 02132, USA.; ^3^ Weill Institute for Neurosciences and Department of Neurology, University of California, San Francisco, San Francisco, CA 94143, USA.

## Abstract

Electrical synapses are expressed ubiquitously across the brain and are crucial components of active neural circuitry and connectomes. Identification of coupled networks in living tissue is limited by technical demands of multiplexed recordings, and no dyes, fluorescent reporters, or genetic labels can now fill the gap. We introduce a novel method of identifying and quantifying electrical synapses, opto-δ*L*, that combines focal photostimulation of soma-targeted opsins with a spike timing–based computation for the strength of electrical synapses to rapidly measure and map electrically coupled networks in vitro. We leverage opto-δ*L* to show that coupled networks of the mature thalamic reticular nucleus extend as far as 100 micrometers, synapse promiscuously across genetic subtypes of neurons, and couple one to four neighboring neurons to each recorded hub cell. We applied opto-δ*L* to cortical networks, where we found that somatostatin neurons were more strongly and prevalently coupled. These results highlight the broad potential of opto-δ*L* to interrogate the identity and roles of electrical synapses in circuitry, behavior, and cognition.

## INTRODUCTION

Chemical and electrical synapses together compose the basis of interneuronal communication across the brain. Neurotransmitter-based chemical synapses underlie communication that is primarily unidirectional and stereotyped in time courses of activation and decay. In contrast, electrical synapses formed by gap junctions (GJs) relay neuronal signals of any size, polarity, and time course bidirectionally between coupled cells, and they do so without the requirement or cost of a spike for signal transduction (*1*, *2*). Most mature mammalian neuronal GJs comprise paired hexameric channels of connexin36 (Cx36) (*3*–*5*), which is expressed widely in GABAergic neurons throughout the brain (*6*). While chemical synapses and the neuronal networks that they form have been described in exquisite detail, descriptions of electrical synaptic transmission and the functions of coupling within networks have been much more limited.

Electrical synapses play diverse roles throughout the brain. They are most widely characterized as mediating synchronization of oscillatory activity in neuronal circuits (*7*–*17*). Beyond synchronization, electrical synapses directly mediate feedforward and lateral excitation and inhibition, reduce signal-to-noise ratio, sharpen coincidence detection, and facilitate object discrimination (*18*–*35*). GJs create offset receptive fields in retinal ganglion cells, where they also contribute to tuning responses, enhanced signal saliency, sensitivity, and enhanced coding of visual features (*18*, *36*–*39*).

Our understanding of the roles that electrical synapses and coupled networks play in the central nervous system is acutely limited by the tools now available. The gold standard for measuring electrical synapses is through paired recordings, in which current is injected into one neuron and a hyperpolarizing deflection can be measured in a coupled neuron (*40*); the ratio of deflections indicates synapse strength. While this method is the most direct, it is time intensive, low throughput, and limited to single synapses in vitro. Only one experiment has successfully recorded from a small sample of coupled neurons, close to the brain surface, in rodents in vivo (*41*). Alternatively, networks can be distinguished by dye-coupling methods (*42*–*49*), but these do not differentiate between primary, secondary, or tertiary connections; fail to quantify the strength of synapses; and are limited to fixed tissue. Updated variations of dye coupling have used exogenously applied molecules that diffuse across a GJ, rendered more specific by limiting entry (*50*) or fluorescence (*51*) to genetically labeled cells. Due to these limitations, electrical synapses and networks remain neglected relative to their chemical counterparts in our evolving understanding of the mammalian brain. Modern connectomes (*52*) have almost entirely neglected electrical synapses, with the exception of the retinal connectome (*53*).

The thalamic reticular nucleus (TRN) is a nucleus of GABAergic neurons (*54*) that provide most of the inhibition to sensory thalamic relay nuclei in rodents and are hypothesized to focus the searchlight of attention upon the sensory surround (*55*). The TRN is composed of distinct neuronal subtypes with spatially and genetically distinct expression (*56*–*58*), and our previous paired recordings indicated that homocellular and heterocellular electrical synapses (coupling matched and disparate cell subtypes) of the TRN (*59*) may provide the only thalamic link between distinct but parallel thalamocortical relay channels (*56*–*58*, *60*). Because visualization of TRN neurons is impeded by myelination of thalamocortical fibers after eye opening, all paired-patch recordings characterizing electrical synapses in TRN have been limited to juvenile tissue (*14*, *59*, *61*–*67*). While Cx36 expression persists into adulthood (*68*) and spikelets have been reported in adults (*69*), the possibility remains that the contributions of GJ networks to TRN function and of interconnectivity within TRN to thalamocortical relay are limited to developmental stages.

Here, we leverage focal photostimulation of a soma-targeted opsin to detect and measure electrical synapses through changes in spike timing in one neuron, which enabled us to map electrically coupled networks in mature TRN and cortex. Our maps in TRN demonstrate that coupled networks comprise both heterocellular and homocellular electrical synapses that range from one to four synapses per hub neuron and extend as far as 100 μm of distance. In cortex, we found that somatostatin-positive neurons couple more frequently than parvalbumin-positive neurons and more strongly than in TRN. In addition to providing a new, functionally relevant and efficient tool for measuring and mapping electrical synapses and networks across the living brain, our results provide insight into the form and function of neural circuits that underpin attentional focus on the sensory surround.

## RESULTS

Precision of neuronal spike timing is a tightly controlled fundamental process (*70*) that is crucial for fidelity of information processing and is often a parameter of interest in investigations of neural coding (*71*–*75*). Electrical synapses are important and bidirectional contributors in determining spike times (*32*, *33*, *63*); a quiescent neuron within a GJ-coupled network delays its neighbors’ spikes by shunting current, while an active neuron shortens spike latencies in neighbors by sending excitatory currents across GJs. Activity in a coupled neighbor can shift spike times of a neuron by tens of milliseconds (*63*). We previously measured changes in spike latencies due to electrical synapses in a cohort of coupled juvenile TRN neurons (*76*) by comparing the means of a set of spike latencies from input in two cases: (i) rheobase stimulation in one neuron alone ($\bar{t_{1}}$) and (ii) rheobase stimulation with simultaneous activation of a coupled neighboring neuron ($\bar{t_{2}}$). We calculated the percentage change in latency of evoked spikes, δ*L*, as

$$\delta L=\frac{100*\left(\bar{t_{1}}-\bar{t_{2}}\right)}{\bar{t_{1}}}$$

This quantity is well correlated with traditional measures of the strength of electrical synapses (*76*). Here, we combined measurements of δ*L* made from a single patched neuron, which we term a “hub” for the network connecting to that neuron, with individual focal photostimulations of nearby neurons expressing the soma-targeted opsin st-ChroME (*77*). Although the δ*L* that we measure in hub neurons results from current flow through GJs from surrounding neurons, the bidirectional nature of GJ-mediated electrical synapses means that activity in hub neurons is also broadcasted to its coupled network. Together, optogenetics and δ*L* measurements thus reduce the requirement for recording an electrical synapse to one patched neuron and simultaneously widely expand the number of potential synapses and networks tested per recording. We refer to this method as opto-δ*L*.

We first validated opto-δ*L* in patched pairs of neurons from juvenile tissue in vitro following injection at postnatal day 0 (P0) of AAV9-CAG-DIO-ChroME-st-P2A-H2B-mRuby. We measured the coupling coefficient (*cc*) for an electrical synapse using the conventional small (50 to 100 pA) hyperpolarizing current injections to one of the neurons (Fig. 1A); *cc* is the ratio of voltage deflections in both neurons in response to current injection into one of the neurons. The example pair in Fig. 1A had mean *cc* of 0.075, close to the average value for coupling between pairs of TRN neurons (*78*). Next, we used a digital mirror device (Mightex Polygon) to deliver one-photon focal (34 to 50 μm^2^) 560-nm photostimulation to cell 2 of this pair, interleaved during trials of rheobase-driven spiking in cell 1, the patched neuron. We used a light intensity minimally sufficient to drive spiking when focused directly on the soma of cell 1 (see Materials and Methods; fig. S1). Because both one-photon and two-photon photostimulation scatter light in live tissue (*79*–*82*), we also measured spike times in cell 1 when photostimulating a blank spot that was equally distant from cell 1 as cell 2 as a control for scatter-induced photoexcitation of cell 1. We computed δ*L* from the latencies of spiking in cell 1 elicited during a set of 10 trials of rheobase stimulation and 10 interleaved trials of rheobase with focal photostimulation of cell 2. Driven by rheobase current (100 pA) alone, cell 1 spiked 176 ± 3 ms after stimulus onset (Fig. 1B, blue traces). During focal photoexcitation of cell 2, cell 1 spiked with a latency of 99 ± 0.3 ms (Fig. 1B, sage traces), and we computed initial δ*L* for the synapse from the two latencies: δ*L* = 60.7 ± 0.7%. During photoexcitation of the equidistant blank spot, cell 1 spiked with latency of 148 ± 3 ms (Fig. 1C, sage traces) and produced δ*L* of 22.7 ± 3.1%. Because δ*L* was significantly larger (by 38.0 ± 3.2%; *P* < 0.01; Friedman’s test followed by Wilcoxon signed-rank test) for photostimulation of cell 2 compared with the blank spot, we concluded that cell 2 was coupled to cell 1, with final corrected δ*L* here taken as 38.0 ± 3.2%. As this example pair demonstrates, it is critical that all opto-δ*L* measurements are normalized by scatter-control measurements, and all opto-δ*L* values reported here are normalized to such controls (see Materials and Methods). Values of opto-δ*L* and *cc* from a cohort of eight pairs were consistent with previous set of measurements of *cc* and δ*L* in which both quantities were measured by direct current injections in juvenile tissue (Fig. 1D) (*76*).

**Fig. 1.**
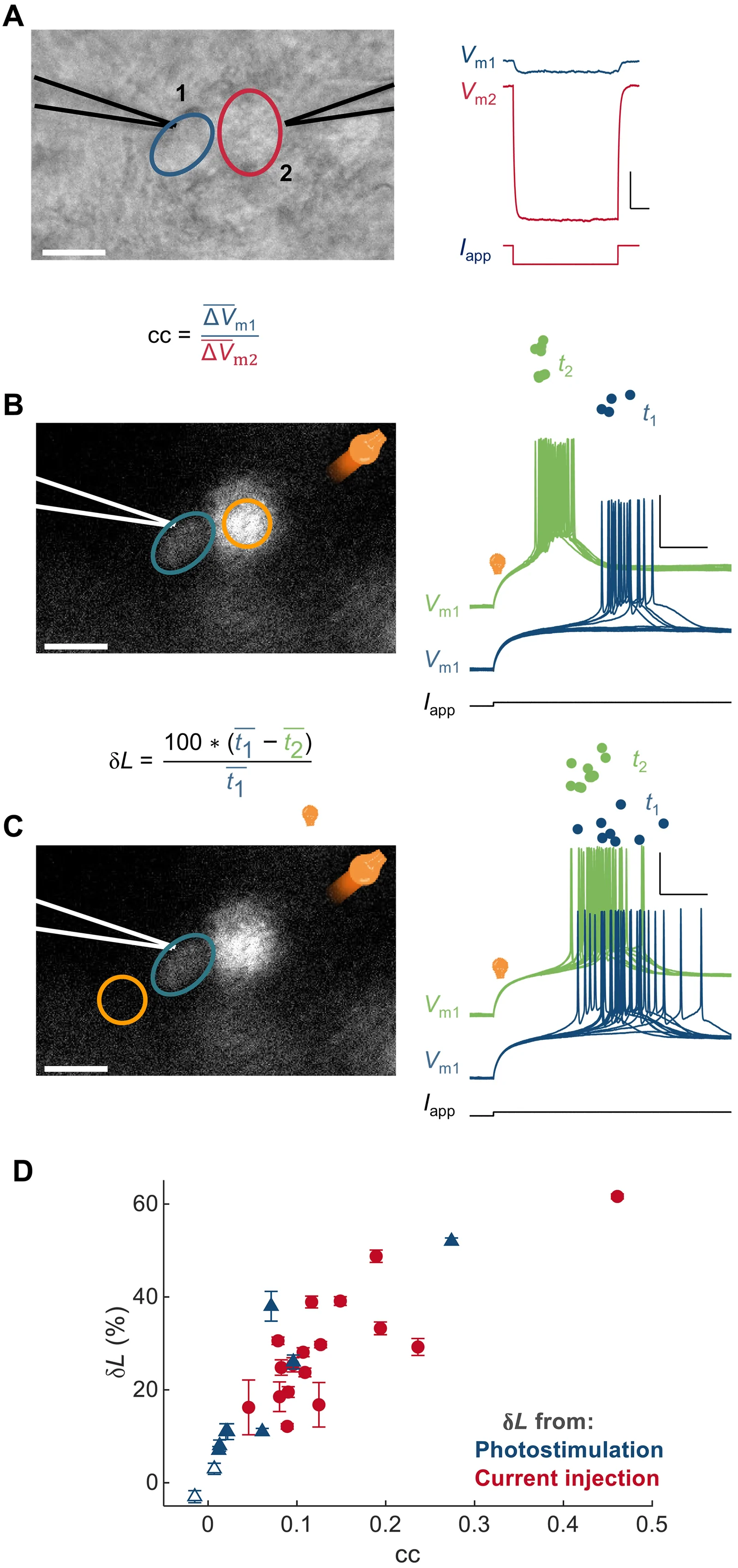
Electrical synapses measured by paired patching and opto-δ*L*. (**A**) Live infrared (IR) image of a pair of juvenile TRN neurons in vitro. Right: Voltage responses (*V*_m_) from both neurons during current injection (*I*_app_) into cell 2. The coupling coefficient (*cc*) is the ratio of voltage deflections in both cells; here, *cc* = 0.075. Scale bar, 100 ms, 2 mV. (**B**) Live fluorescence (st-ChroME-mRuby) image of the same pair from (A). Orange circle: location and area of focal photostimulation of cell 2. Scale bar: 12.5 μm. Right: Spiking in neuron 1 for rheobase (100 pA) applied current alone (blue) and during simultaneous focal photostimulation of neuron 2 (sage). Dots: first spike for each trial. Scale bars: 50 ms, 25 mV. Initial δ*L* calculated from the difference in mean spike latencies: 60.7 ± 0.7%. (**C**) Orange circle: location and area of focal photostimulation of an equidistant blank spot. Right: Spiking in neuron 1 for 110 pA applied current alone (blue), and with simultaneous photostimulation of the blank spot (sage). Dots: first spikes in each trial. Scale bars: 20 ms, 25 mV. Blank spot δ*L*: 22.7 ± 3.1%. Corrected δL for this pair: 38.0 ± 3.2% (*P* < 0.01, Wilcoxon signed-rank test). (**D**) Red: pairs with *cc* and δ*L* both measured by current injection. Blue: pairs with *cc* measured by current injection and δ*L* measured by photostimulation. Open symbols: δ*L* not significant. Red points adapted (averaged for each cell) from Haas (*76*). Licensed under CC BY 4.0.

We further validated opto-δ*L* using PV-Cre-Cas9 mice injected with AAV5-sgRNA-Cx36-BFP in TRN to knock down Cx36, which expresses in neurons (Fig. 2A). As a control for nonspecific genome abscission, we used AAV5-sgRNA-Cx29-BFP to knock down Cx29 in TRN, which only expresses in glia, where Cas9 is not expressed. In six slices prepared from five Cx36 KD mice, we detected one electrical synapse that survived knockdown (KD) (δ*L* = 41.0 ± 2.0%) among 7 patched hub neurons and 36 neighboring neurons from six slices and five mice using opto-δ*L* (Fig. 2B; mean uncoupled δ*L* = 0.2 ± 0.6%). The patched hub neuron with a synapse exhibited spikelets, additional evidence of a true electrical synapse rather than a falsely positive opto-δ*L*. In contrast, we detected 12 electrical synapses by opto-δ*L* between 5 patched hub neurons and 34 neighboring neurons in three slices from two Cx29 KD mice (Fig. 2C; mean δ*L* = 24.7 ± 6.5%, with coupling detected in slices from both mice; mean uncoupled δ*L* = 0.0 ± 1.0%). Coupling incidence was lower in the Cx36 KD (2.8%) compared with that in the Cx29 KD control (35.3%; *P* < 0.01, Fisher’s exact test). Collectively, these measurements validate the use of a single electrode combined with focal photostimulation of soma-targeted opsins to identify and characterize electrical synapses. Further, these validations provided a foundation for using opto-δ*L* to identify and measure electrical synapses and networks in mature TRN tissue, where spikelets that appear in depolarized neurons provide evidence of electrical synapses (Fig. 3) (*69*) but where heavy myelination precludes paired recordings.

**Fig. 2.**
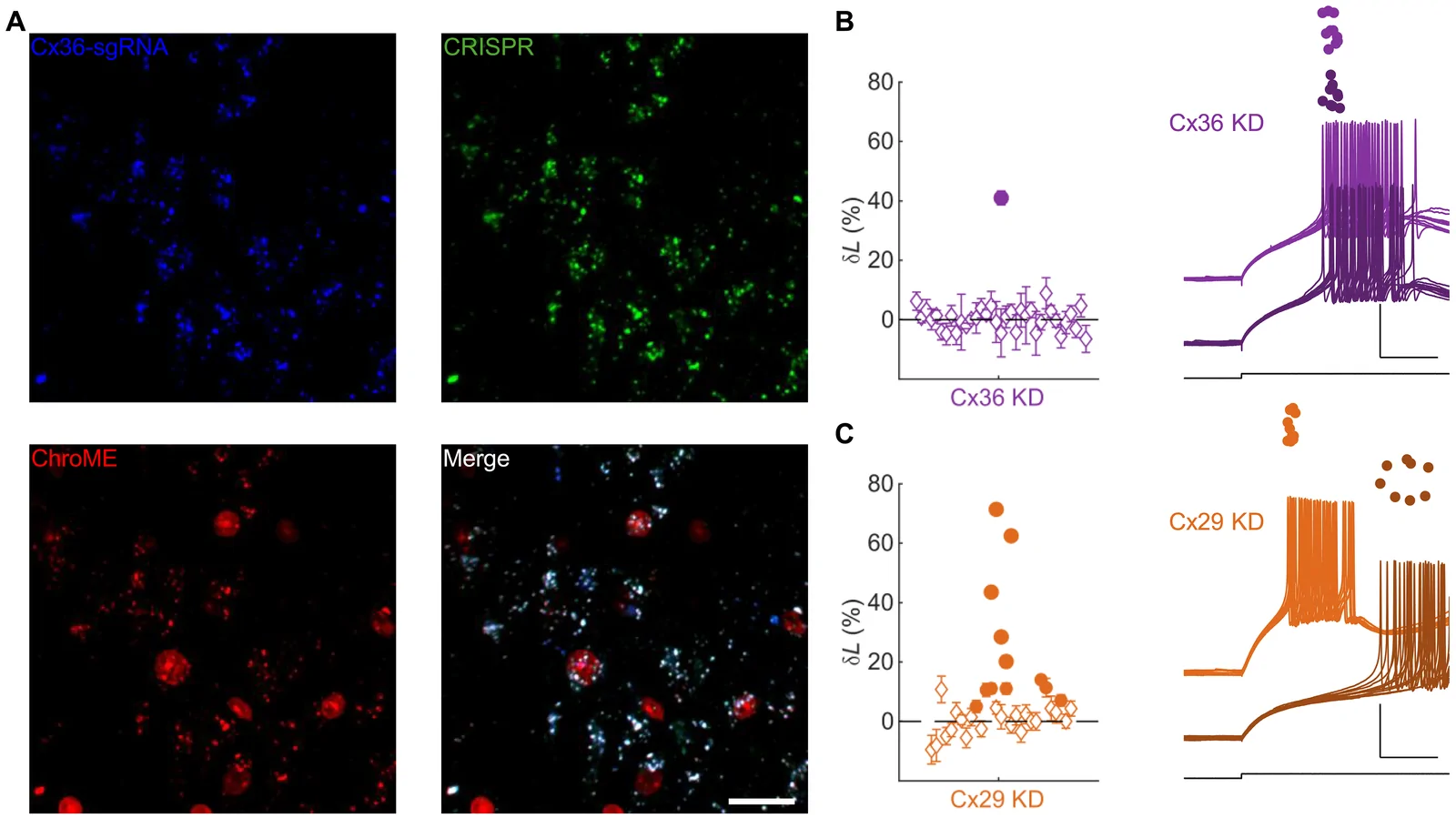
Connexin knockdown in TRN. (**A**) Maximum intensity projections of TRN tissue following CRISPR knockdown (KD) of Cx36 (Cx36 KD). Blue: Cx36-sgRNA-BFP; green: PV-Cre-Cas9/GFP; red: ChroME-mRuby. Scale bar: 20 μm. (**B**) Left: Measurements of δ*L* in TRN neurons following Cx36 KD (*n* = 36 tested connections to seven patched neurons from six slices from five mice). δ*L* for one tested synapse (filled circle, δ*L* = 41.0 ± 2%) was significant; 35 tested connections were not significant (mean δ*L* = 0.2 ± 0.6%; *P* > 0.05, Wilcoxon signed-rank test). Right: Example of spiking responses used to measure δ*L* in Cx36 KD tissue. Darker traces: responses during control trials; lighter traces: responses during focal target cell photostimulation. Scale bars: 25 mV, 25 ms. (**C**) Left: Measurements of δ*L* in TRN neurons following Cx29 KD (*n* = 34 tested connections to five patched neurons from three slices from two mice). Filled circles: significant δ*L* values (*n* = 12; mean δ*L* = 24.7 ± 6.5%); open diamonds: δ*L* not significant (*n* = 22; mean δ*L* = 0.0 ± 1.0%; *P* > 0.05, Wilcoxon signed-rank test). Connections in Cx29 KD were more frequent than in Cx36 KD (*P* < 0.01, Fisher’s exact test). Right: Example of spiking responses used to measure δ*L* in Cx29 KD tissue. Darker traces: responses during control trials; lighter traces: responses during focal target cell photostimulation. Scale bars: 25 mV, 25 ms.

**Fig. 3.**
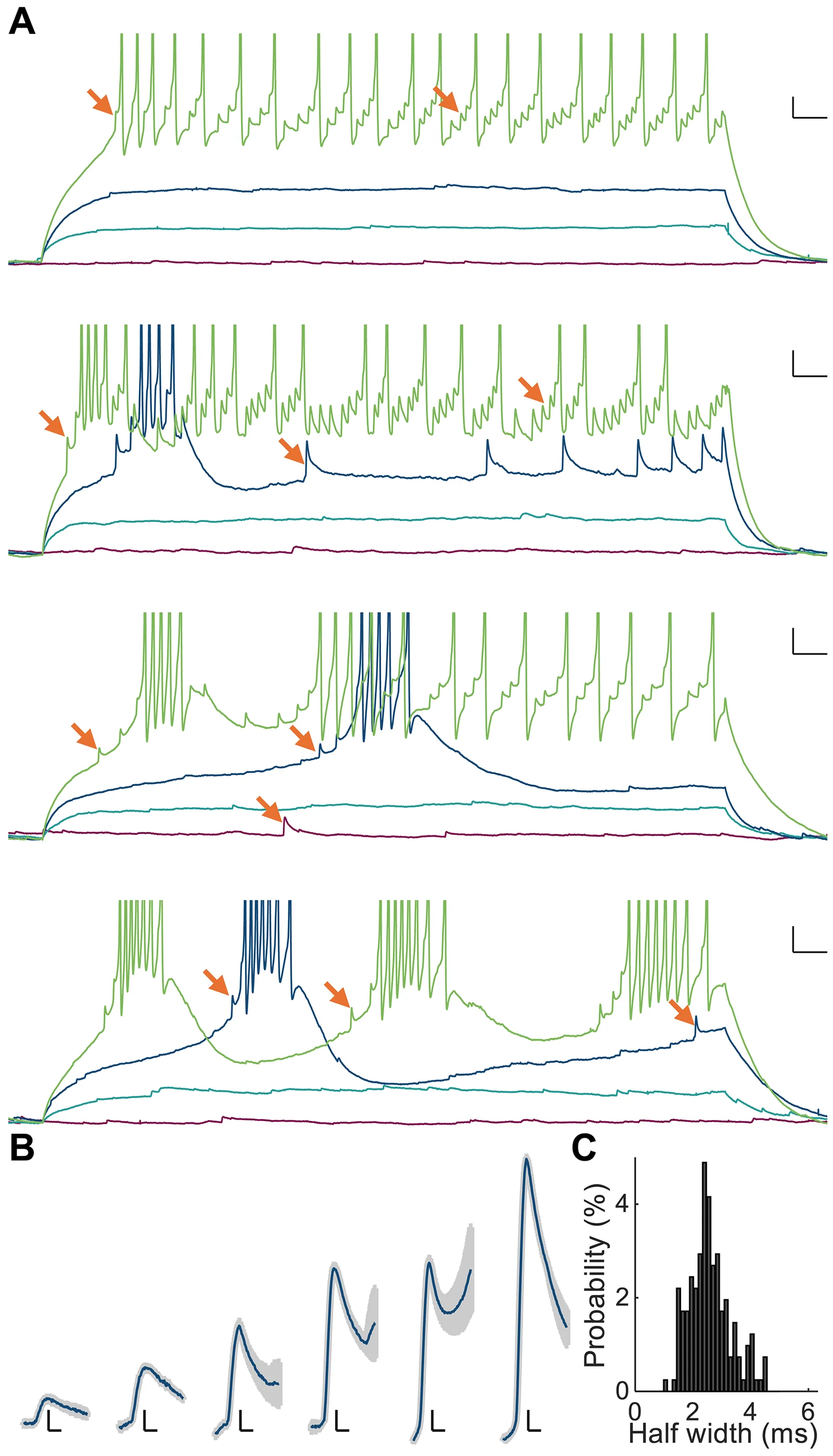
Spikelets provide evidence of electrical synapses in adult TRN. (**A**) In four example TRN cells from adult tissue, spikelets (arrows) appeared upon injection of depolarizing current (50, 100, and 150 pA from *V*_m_ = −70 mV) that presumably also excited an unrecorded coupled neuron. Spikelets are readily distinguished from sEPSPs by repeated amplitudes, patterned arrival times, and short duration. We detected spikelets in 16 of 138 tested neurons. Scale bars: 5 mV, 25 ms. Spikes were truncated for display. (**B**) Peak-aligned averages (±SEM) of spikelets from six example neurons. Scale bars: 0.5 mV, 1 ms. (**C**) Distribution of spikelet half-width (mean, 2.60 ± 0.72 ms) from 158 spikelets from 16 neurons.

Genetically and anatomically distinct sets of neurons form distinct sets of relay pathways through the thalamocortical system (*56*–*58*, *60*); as GABAergic responses within TRN do not result from optogenetic stimulation of TRN neurons in adult tissue (*83*), electrical synapses within the TRN may be the only thalamic link between these information channels. We previously used paired recordings in juvenile tissue to test whether TRN electrical synapses interconnect neurons of distinct cell types (*59*). Those experiments necessitated the use of crossed Cre reporter mice to label neurons for juvenile recordings, presenting an additional possible confound of mislabeling neurons due to Cre off-target recombination (*84*). Therefore, we used opto-δ*L* to identify, measure, and characterize homocellular and heterocellular electrically coupled networks in mature TRN in vitro, at least 2 weeks after injection of AAV9-CAG-DIO-ChroME-st-P2A-H2B-mRuby into the TRN of SOM-Cre and PV-Cre mice at ages P28 to P35. To map homocellular electrical synapses and networks, we patched ChroME-expressing mRuby^+^ hub neurons (SOM^+^) in slices from injected SOM-Cre mice and measured δ*L* induced by focally photostimulating other nearby mRuby^+^ neurons (Fig. 4, A to C, and fig. S2). To map heterocellular electrical synapses and networks, we patched mRuby^−^ neurons (SOM^−^) in slices from injected SOM-Cre mice and measured δ*L* induced by focally photostimulating nearby mRuby^+^ neurons (Fig. 4, D to F, and fig. S2). We also measured homocellular networks among PV^+^ neurons in slices from injected PV-Cre mice (Fig. 4, G to I, and fig. S2). To compensate for excitation from scatter in these experiments, we used correction procedures for each hub neuron (fig. S3; see Materials and Methods); scatter effects depend on factors including light intensity, opsin expression, and input resistance, as well as distance. Depolarization from scatter diminishes to insignificant levels by 50 to 100 μm from the photostimulated area (fig. S4). We note that mapping heterocellular networks has the advantage of targeting opsin-lacking neurons for recording, thereby averting the need for compensation in those experiments. Opto-δ*L* can also be measured using two-photon photostimulation (fig. S5).

**Fig. 4.**
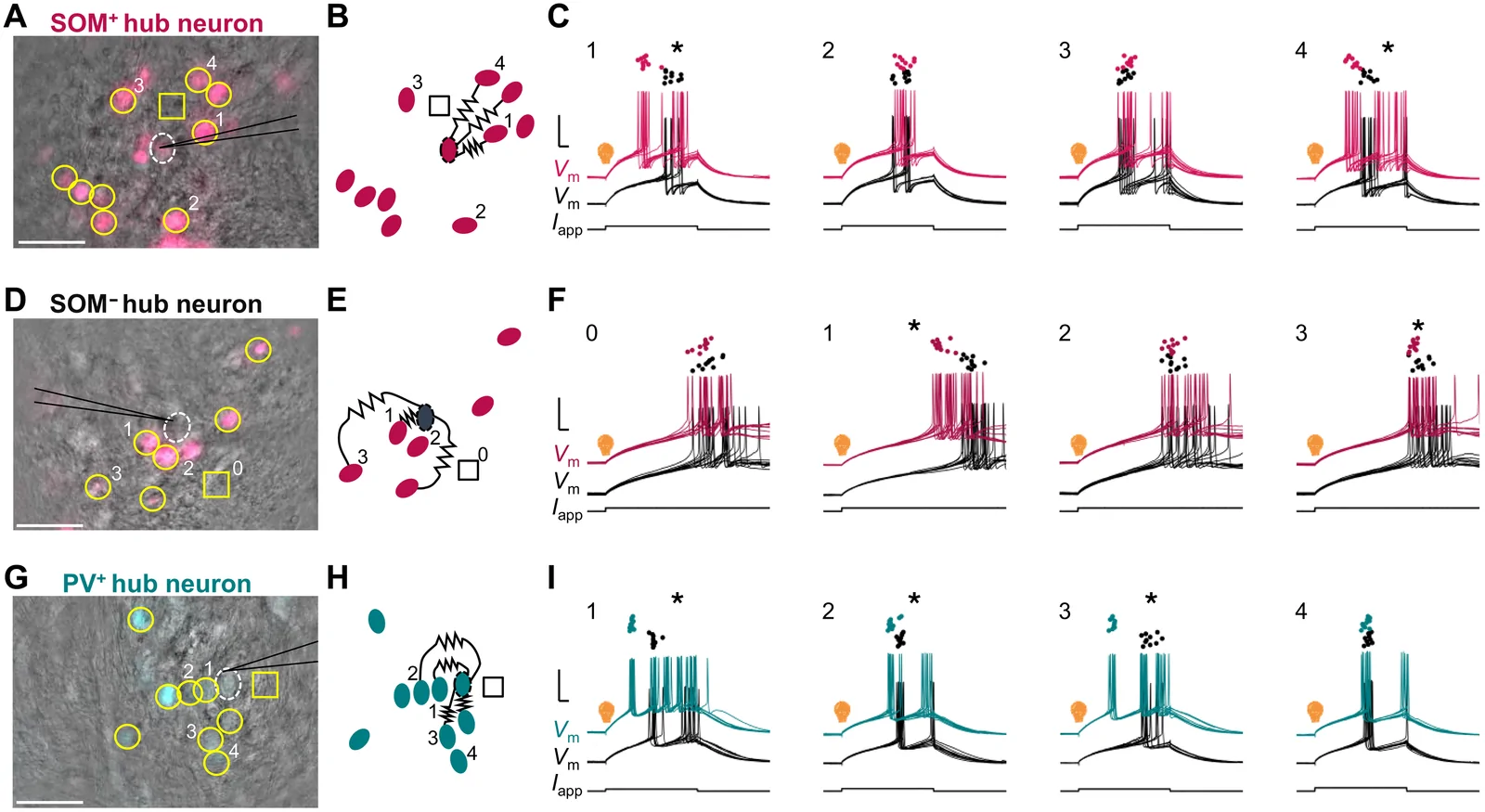
Mapping coupled networks between TRN neurons. (**A**) Overlay of IR and fluorescence images from live recording of st-ChroME-mRuby–expressing (pseudocolored magenta) SOM^+^ TRN neurons. White dashed circle: patched hub neuron. Yellow circles: focally photostimulated cells. Square: empty control area. Scale bar: 50 μm. (**B**) Diagram of the coupled network embedded within all tested neurons from (A). (**C**) Spiking responses from opto-δ*L* measurements from numbered cells in (A). Dots: first spikes from each trial. Black traces: responses during rheobase-only trials; colored traces: responses during added focal photostimulation of the numbered cell. Traces vertically offset for clarity. Scale bars: 5 ms, 20 mV. Asterisks: *P* < 0.05 (Wilcoxon signed-rank test). (**D** to **F**) Representative heterocellular network among SOM^−^ and SOM^+^ neurons, as described for (A) to (C). (**G** to **I**) Example homocellular network among PV^+^ neurons, as described for (A) to (C).

To characterize mature TRN networks, we used opto-δ*L* to test connections to 95 patched hub neurons (mean *R*_in_, 146.5 ± 6.5 MΩ; and mean resting *V*_m_, −67.5 ± 0.7 mV) from 22 slices taken from 21 PV-Cre mice and 44 slices taken from 42 SOM-Cre mice injected with AAV9-CAG-DIO-ChroME-st-P2A-H2B-mRuby into the TRN, ultimately testing 501 connections between hub neurons and neighboring neurons that revealed 39 networks (21 SOM^+^, 4 SOM^−^/SOM^+^, and 14 PV^+^). For each hub neuron, we tested an average of 6.1 neighboring neurons for electrical synapses; the largest network tested was 20 neighboring neurons. Overall, we measured 83 electrical synapses via opto-δ*L* across all combinations of genetic subtypes tested (Fig. 5A).

**Fig. 5.**
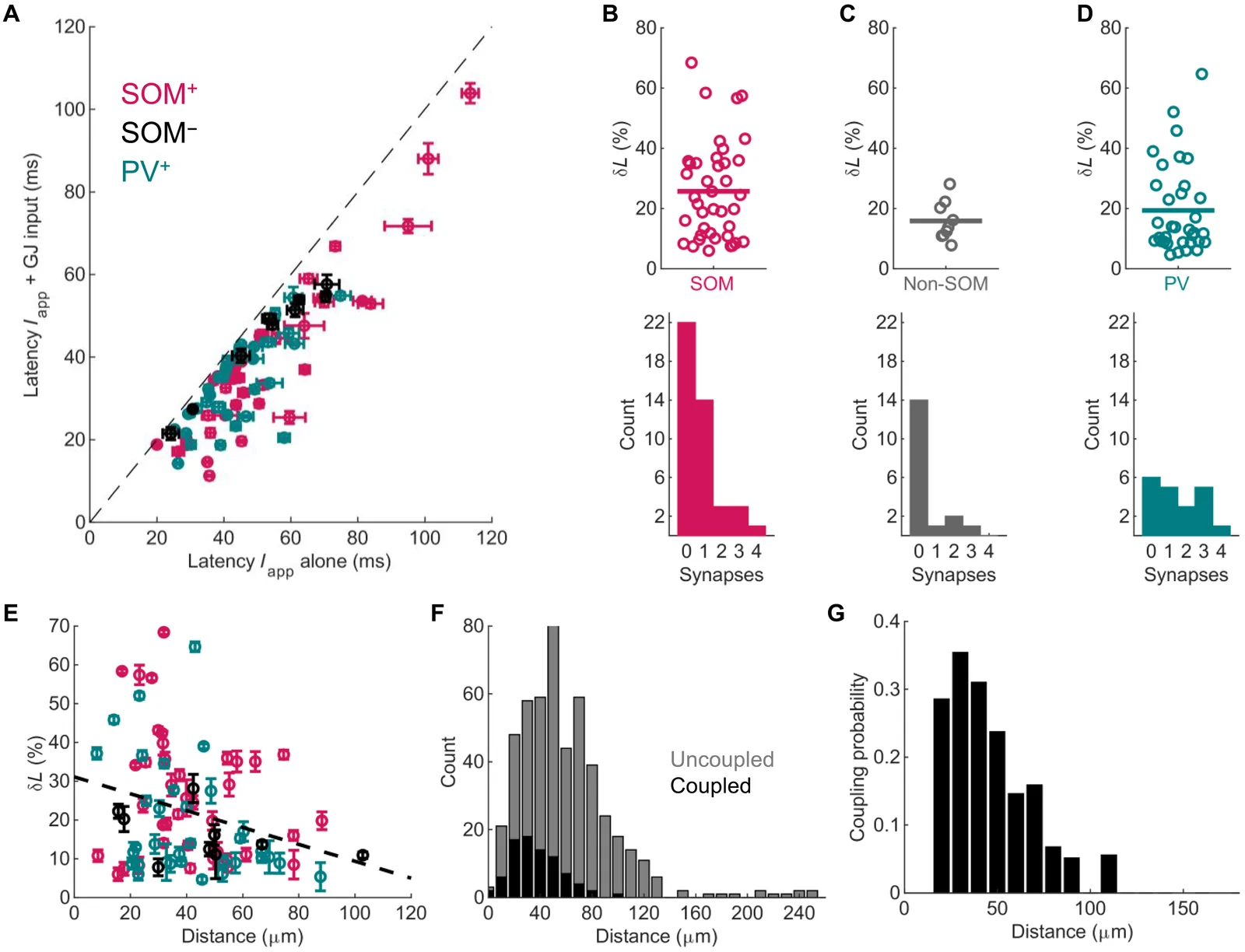
Distribution of TRN electrical synapses and networks over strength and space. (**A**) Latencies (±SEM) used to compute δ*L* for 40 SOM^+^, 9 SOM^−^, and 34 PV^+^ synapses. (**B**) Top: Summary of δ*L* for homocellular SOM^+^ synapses (mean δ*L* = 25.6 ± 2.6%; *n* = 40 synapses across 21 networks in 44 slices from 42 mice). Bottom: Distribution of network sizes (number of coupled neighbors per hub cell) for SOM^+^/SOM^+^ networks (mean size, 1.6 ± 0.04 synapses per network). (**C**) As in (B) for heterocellular SOM^−^/SOM^+^ synapses (mean δ*L* =15.9 ± 2.2%; *n* = 9 synapses across four networks in five slices from five mice; mean size, 2.0 ± 0.2 synapses per network). (**D**) As in (B) for homocellular PV^+^ synapses (mean δ*L* = 19.4 ± 2.6%; *n* = 34 synapses across 14 networks in 22 slices from 21 mice; mean size, 2.1 ± 0.1 synapses per network). (**E**) Distribution of δ*L* across intersomatic distance for each subtype in TRN (*n* = 83 synapses). (**F**) Number of photostimulated neurons that were (black, *n* = 83) or were not (gray, *n* = 418) coupled to a patched hub neuron, plotted against distance from the hub neuron (*n* = 501 total connections tested). (**G**) Probability of coupling across intersomatic distance.

Starting from 51 SOM^+^ hub neurons (mean *R*_in_, 166.7 ± 9.8 MΩ; mean resting *V*_m_, −68 ± 1.0 mV; and mean rheobase stimulus, 51.4 ± 4.5 pA), we found 40 synapses between SOM^+^ hub neurons and SOM^+^ neighbors, ultimately mapping 21 homocellular networks in 44 slices from 42 mice. The mean coupling incidence was 16.0% across all tested SOM^+^ connections (*n* = 250 connections tested). Some TRN cells appear to lack Cx36 expression (*61*); limiting our computation of coupling incidence to hub cells that had at least one synapse, we found coupling incidence was 41.7% (*n* = 96 tested connections). The mean δ*L* between SOM^+^ neurons was 25.7 ± 2.6%, and the mean homocellular SOM network contained 1.6 ± 0.04 electrical synapses per hub neuron (Fig. 5B).

To identify and map heterocellular networks, we patched 20 SOM^−^ hub neurons (mean *R*_in_, 118.4 ± 10.7 MΩ; mean resting *V*_m_, −68 ± 1.5 mV; mean rheobase stimulus, 92.9 ± 13.0 pA) and photostimulated neighboring SOM^+^ neurons; we measured nine connections with significant values of δ*L*, ultimately mapping four heterocellular networks in 18 slices from 16 mice. The mean coupling rate was 7.3% over all 123 connections tested and 28.1% across 32 tested connections from a hub cell with at least one connection. The mean δ*L* for SOM^−^ to SOM^+^ connections was 15.9 ± 2.2%, and the mean heterocellular network contained 2.0 ± 0.2 electrical synapses per hub neuron (Fig. 5C).

To measure homocellular networks among PV^+^ neurons, we recorded from 24 PV^+^ hub neurons (mean *R*_in_, 127.1 ± 9.4 MΩ; mean resting *V*_m_, −66 ± 1.3 mV; mean rheobase stimulus, 50.7 ± 13.3 pA) and measured 34 synapses using opto-δ*L*, ultimately mapping 14 homocellular networks in 22 slices from 21 mice. The mean coupling rate was 28.8% across all 128 tested connections and 51.5% from hub cells with least one electrical synapse (*n* = 66 connections tested). The mean δ*L* for PV^+^ to PV^+^ electrical synapses was 19.4 ± 2.6%, and the mean network size was 2.1 ± 0.1 direct electrical synapses per hub neuron (Fig. 5D).

Across TRN networks, there were no significant differences in average δ*L* between subtypes (Fig. 5, B to D; *P* > 0.05). The mean δ*L* across all subtypes was 22.6 ± 1.6% (*n* = 83 synapses), and 50% of synapses had δ*L* between 10.5 and 34.4%. The largest δ*L* was 68.4%. PV^+^ neurons were coupled more frequently (28.8%) than either SOM^+^ neurons (16.0%; *P* = 0.04, Fisher’s exact test) or SOM^−^ neurons were coupled to SOM^+^ neurons (7.3%; *P* < 0.01, Fisher’s exact test), and SOM^+^ neurons were coupled to other SOM^+^ more frequently than SOM^−^ neurons (*P* = 0.02, Fisher’s exact test). We found no electrical synapses for some hub cells. We detected no electrical synapses for 30.0% of PV^+^ hub neurons, 51.2% of SOM^+^ hub neurons, and 77.8% of SOM^−^ hub neurons. It was rarer to find no electrical synapses from a PV^+^ hub neuron compared with a SOM^−^ hub neuron (*P* = 0.01, Fisher’s exact test), but the difference was not significant for comparisons between other cell types (*P* > 0.05, Fisher’s exact test). The average intersomatic distance between all coupled TRN neurons was 41.7 ± 2.2 μm (*n* = 83 synapses). There were no differences in average intersomatic distances between coupled subtypes (SOM^+^, 41.3 ± 2.9 μm; SOM^−^, 47.1 ± 8.9 μm; and PV^+^, 40.7 ± 3.2 μm; *P* > 0.05, Mann-Whitney *U* test).

Opto-δ*L* allowed us to expand sampling of the spatial extent of coupling from each TRN hub neuron from previous paired-patching work. Measuring electrical synapses by patching pairs introduces spatial selection biases to the pairs that are ultimately tested in experiments; this effect may have contributed to a discrepancy between results from rat TRN, suggesting that single electrical synapses occur only within intersomatic distances less than 40 μm (*78*), and results from dye-coupling, which permits spread of dye over several synapses, indicating a median intersomatic distance of 80 to 120 μm (*67*). We leveraged the ability of opto-δ*L* to stimulate distant neurons, testing synapses up to 250 μm. Across all neuron types grouped together, we report that TRN neurons that were closer together showed larger values of δ*L* (Fig. 5E; correlation coefficient *r* = −0.28, *P* = 0.01, Pearson’s correlation) and were more likely to produce significant δ*L* (Fig. 5, F and G). As measured by δ*L*, neurons within 50 μm were coupled more frequently (42.5%) compared with neurons between 50 and 100 μm (11.3%; *P* < 0.01, Fisher’s exact test). These results confirm previous reports that coupling diminishes with intersomatic distance. Collectively, our data also showed that electrically coupled TRN networks for all cell types link neurons as far as 100 μm apart (Fig. 5, E to G).

To extend opto-δ*L* as a tool for measuring and mapping coupled networks across the brain, we used opto-δ*L* to map coupled PV^+^ and SOM^+^ networks in primary somatosensory cortex. In five slices taken from three mice expressing st-ChroME in SOM^+^ neurons, we tested a total of 15 connections from five patched hub cells (mean *R*_in_, 186.8 ± 34.4 MΩ; mean resting *V*_m_, −56.7 ± 2.6 mV; and mean rheobase stimulus, 65.6 ± 17.0 pA). One example SOM^+^ cortical network comprised three neurons with δ*L* values of 63.0 ± 1.1%, 35.3 ± 5.0%, and 55.8 ± 1.1% (Fig. 6, A to C). In seven slices taken from six mice expressing st-ChroME in PV^+^ neurons, we tested 32 connections from eight hub cells (mean *R*_in_, 92.3 ± 21.0 MΩ; mean resting *V*_m_, −63.4 ± 1.0 mV; and mean rheobase stimulus, 73.1 ± 6.4 pA). A representative map that we identified for a PV^+^ cortical network consists of only one weak connection with δ*L* of 9.0 ± 0.6 (Fig. 6, D to F). We identified a total of six synapses among SOM^+^ neurons (Fig. 6G; mean δ*L* = 44.8 ± 5.6%) and three synapses among PV^+^ neurons (Fig. 6G; mean δ*L* = 29.5 ± 18.9%). As in TRN, coupling strength in cortex diminished with intersomatic distance (Fig. 6H). In cortex, inhibitory PV^+^ or SOM^+^ cells are a minority population that is interspersed between excitatory principal cells, resulting in sparser sampling of smaller intersomatic distances (Fig. 6I), but the average intersomatic distance between coupled TRN neurons (41.7 ± 2.2 μm, *n* = 83 synapses) as measured by δ*L* was not different from that in coupled cortical neurons (55.8 ± 8.6 μm, *n* = 9 synapses; *P* = 0.14, Mann-Whitney *U* test). Also similar to TRN, we found coupling in cortex at distances exceeding 100 μm (Fig. 6J). However, in contrast, our data show that mean coupling strength δ*L* among SOM^+^ neurons was 46.7% larger in cortex than mean δ*L* in TRN (*P* = 0.03, Mann-Whitney *U* test). Further, we noted that PV^+^ coupling incidence in cortex (9.4%) was lower than for SOM^+^ (40.0%; *P* = 0.02, Fisher’s exact test), contrary to the pattern in TRN where PV^+^ coupling was most frequent. These differences offer evidence that the strength, arrangements and incidence of electrical coupling between neurons may be tailored to the unique requirements of each brain area in which they appear.

**Fig. 6.**
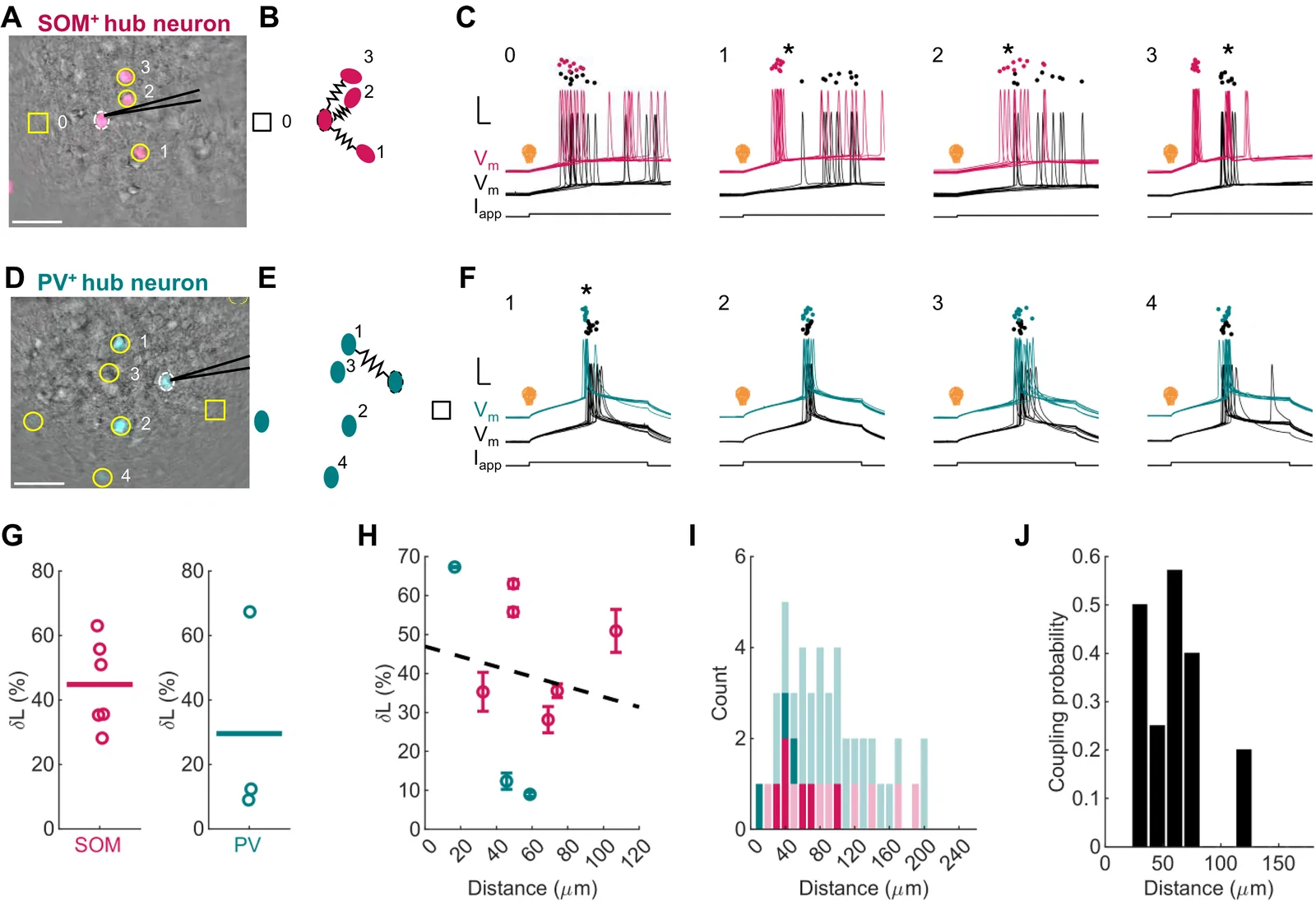
Coupled networks in cortex. (**A**) Overlay of IR and fluorescence images from live recording of st-ChroME-mRuby–expressing (pseudocolored magenta) SOM^+^ somatosensory cortical neurons. White dashed circle: hub neuron. Yellow circles: focally photostimulated cells. Square: empty control area. Scale bar: 50 μm. (**B**) Diagram of the coupled network determined from all tested connections in (A). (**C**) Spiking responses from opto-δ*L* measurements from numbered cells in (A). Dots: first spikes from each trial. Black traces: responses during rheobase-only trials; colored traces: responses during added focal photostimulation of the numbered cell. Traces vertically offset for clarity. Scale bars: 5 ms, 20 mV. Asterisks: *P* < 0.05 (Wilcoxon signed-rank test). (**D** to **F**) Example network among cortical PV^+^ neurons, as described for (A) to (C). Six additional more-distant neurons without electrical synapses were also tested for this hub neuron (not shown). (**G**) Left: Summary of δ*L* for homocellular SOM^+^ cortical synapses (mean δ*L*, 44.8 ± 5.6%; *n* = 6 synapses in three slices from three mice). Right: Summary of δ*L* for homocellular PV^+^ synapses (mean δ*L*, 29.5 ± 18.9%; *n* = 3 synapses in three slices from three mice). (**H**) Distribution of δ*L* across intersomatic distance for each subtype in cortex. (**I**) Number of photostimulated neurons that were (darker, *n* = 9) or were not (lighter, *n* = 38) coupled to a patched hub neuron, plotted against distance from the hub neuron (*n* = 47 total connections tested). (**J**) Probability of coupling across intersomatic distance.

## DISCUSSION

Cx36 is widely expressed throughout the mature mammalian brain, yet functional measurements and maps of electrically coupled networks remain absent in live tissue. Here, we introduce a novel method to map networks in living tissue, opto-δ*L*, which leverages focal photostimulation of soma-targeted opsin-expressing neurons combined with functional measurements of changes in spike timing, including tests of significance, that result from the excitation that is shared across electrical synapses. Using opto-δ*L*, we provide direct evidence that functional electrical synapses in TRN persist into adulthood, which has previously been limited to indirect evidence of spikelets (*69*) and Cx36 expression (*68*). We also demonstrate that adult GABAergic TRN neurons of differing molecular subtypes are coupled in homocellular and heterocellular electrical networks and extend further across tissue than previously identified by dual-cell patching (*78*). Further, we mapped networks of direct electrical connections in TRN and revealed that these networks are limited to far fewer neurons than dye coupling had previously suggested (*67*). These results imply that the electrical synapses and networks of the mature TRN provide a link between thalamocortical pathways and that they do so by forming networks that are selective in size.

In TRN, we observed via δ*L* that PV^+^ neurons formed electrical synapses more frequently than among SOM^+^ neurons (Fig. 6B). We hypothesize that is because PV expression is more of an umbrella than a distinct marker for a subset of TRN neurons, consistent with previous descriptions (*57*, *58*, *83*, *85*–*88*), and, therefore, our maps of PV^+^ neurons might include both heterocellular and homocellular electrical synapses. However, other experiments suggest that PV^+^ neurons are a distinct subset of TRN neurons that primarily form connections with first-order thalamus (*56*, *89*), similar to subsets identified by the calcium-binding protein CB (*58*) and the transcription factor SPP1 (*57*), and that they are distinct from SOM^+^ neurons. If the latter were the case, our results imply that TRN neurons that project to first-order thalamus form more electrical synapses than SOM^+^ neurons. In turn, that difference would imply that coordination between larger numbers of TRN neurons is more prevalent within primary thalamic relay circuits.

The electrical connections that we observed as networks emanating from one neuron were small, ranging from one to four electrical synapses; δ*L* provides the first direct measurements of the number of neurons composing a functionally coupled network. A previous dye-coupling study in juvenile TRN stained networks with an average of 7 neurons and as large as 10 to 20 neurons (*67*). Dye-coupling allows spread of dye between neurons that are not directly coupled to the originally patched neuron and thereby less likely to influence its spike times. In contrast, opto-δ*L* is likely to detect only direct connections, as it is quite unlikely that an indirectly coupled neuron would affect spike timing by the magnitudes that we regularly observed. As a result, networks defined by opto-δ*L* measurements provide functional evidence of the synaptic inputs that are relevant to network function, compared with networks defined by dye coupling. The overall proportion of neurons that were coupled using latency measurements was 16.6%, which is less than that reported from paired recordings in juvenile rats and mice, where reports vary between 31% (*61*), 45% (*59*), and 71% (*78*). There are several possible explanations for this discrepancy. The most likely cause for this difference is that our tests and maps of δ*L* included TRN neurons that were farther away from the patched neuron, whereas previous paired-patching experiments often select for neurons that are much closer together. Species differences or decreases in coupling frequency from juveniles to adults could also contribute to the discrepancy. Our observations of networks composed of one to four local neurons are consistent with results inferring that TRN neurons form small clusters of coupled neurons (*78*). However, opto-δ*L* revealed coupling that extends further than previous evidence from paired recordings (*78*), increasing the options for communication between functionally distinct thalamocortical channels. We interpret the small sizes of these networks to indicate that electrical coupling and network membership are more selective and targeted than ubiquitous or promiscuous. The full extent of networks beyond single hub neurons, the specific rules that determine connectivity, and the functional outcomes for thalamocortical processing remain open and important questions.

Opto-δ*L* opens possibilities for more easily detecting and measuring coupled networks throughout the brain. Our findings from our use of opto-δ*L* show that PV^+^ neurons are more sparsely coupled than SOM^+^ neurons in cortex, differing from TRN, and that SOM^+^ coupling produces larger values of δ*L* in cortex and suggesting that electrical networks are tailored to each brain area in which they operate. In most brain areas, an additional barrier to identifying and measuring coupling by paired patching is the need to visually identify GABAergic neurons. Use of fluorescent labels and opto-δ*L* mitigates both of these barriers, and dual or triple fluorophore expression will expand the possibilities further. Measurements of δ*L* can in principle be used in vivo with focal holographic stimulation (*77*, *90*) to map coupled networks, and we suggest that this would be a valuable next step to map networks fully in three dimensions. Combining opto-δ*L* with activity indicators may reveal how activity propagates through a network under varied conditions. Further, one could examine how network membership and dynamics change in response to different plasticity paradigms.

Our experiments likely undersampled electrical synapses and networks for several reasons. First, slicing brain tissue results in neuron loss and cuts connections. Second, our previous experiments determined that heterocellular synapses are common (*59*), but we were unable to photostimulate neurons that did not express opsin, which represent another pool of possibly undetected synapses. Failure of virus transfection could be a source of undersampling in our experiments. Furthermore, due to finite recording times, we only tested a subset of nearby neurons for each hub neuron. Our method may also underestimate connections due to a floor effect from our light scatter compensation and differences in sensitivity between *cc* and δ*L* for detecting very weak synapses. A floor effect would be more pronounced for neurons with lower input resistance, as is also the case for *cc*. While our experiments failed to detect any electrical synapses for 42 neurons, we expect that we overestimated the number of TRN neurons without electrical synapses. It is possible that some TRN neurons fail to form electrical synapses, which would be consistent with the observation that some TRN neurons do not stain for Cx36 (*61*). Likewise, we interpret our report of one to four electrical synapses for TRN networks as establishing a floor for network size.

Both opto-δ*L* and *cc* have inherent limitations. The measures are correlated (Fig. 1D), but, due to the nonlinearities of spike generation, there is no simple translation between the two quantities. Measurement of *cc* is modulated by subthreshold nonlinearities, which can be amplified by as much as a factor of 4 by the persistent sodium current (*14*, *91*, *92*) and is similarly modulated by other voltage-dependent conductances that are active near rest (*93*–*96*) [reviewed by Pereda *et al.* (*97*)]. By the same tokens, *cc* is inexorably intertwined with resting membrane potential, the amplitude of injected current used to measure it, and changes with drift or any other factor that alters input resistance. Electrical synapses often express full or partial rectification; *cc* detects rectification readily, whereas opto-δ*L* would require separately patching both neurons of an identified pair and taking care to match initial latencies to measure asymmetry. In addition to light scatter (see Materials and Methods), incomplete soma limitation of opsins, although reported to be modest (*77*, *98*), could add error to soma-proximal opto-δ*L* measurements that could be mitigated by use of cell-filling dyes. Like *cc*, opto-δ*L* is also sensitive to resting membrane potential, subthreshold nonlinearities, and the magnitude of spike-driving currents used (*76*) and as a result could, in principle, vary between cell types. Smaller amounts of current that yield correspondingly longer initial latencies allow for more robust measurements, but spiking precision diminishes as one approaches spiking threshold. Therefore, current application above rheobase, but only enough so to drive reliable spikes, is ideal for δ*L* measurements. In comparing *cc* and opto-δ*L*, it must be noted that opto-δ*L* clarifies the functional impact of an electrical synapse; that is, electrical synapses alter spike times in the neurons, circuits, and networks that they couple.

## MATERIALS AND METHODS

### Animals

Experiments were performed as approved by the Lehigh University IACUC protocol no. 255. SOM-Cre (Jax, 013044) or PV-Cre (Jax, 008069) mice were used for juvenile and all adult experiments; one Calbindin-Cre (Jax, 028532) mouse was used for one juvenile experiment (Fig. 1). Mature mice were at least P28 for surgeries and P42 for electrophysiological recordings. To target our CRISPR KD selectively to the PV-expressing GABAergic neurons, we crossed male Rosa26-lox-stop-lox-Cas9/green fluorescent protein (GFP) mice (Jax, 026175) with PV-Cre (Jax, 017320), generating Cas9 and GFP reporter expression in PV neurons (PV-Cre-CRISPRCas9-GFP) (*99*).

### Electrophysiology

Mice were transcardially perfused with *N*-methyl-d-glucamine (NMDG) artificial cerebrospinal fluid (ACSF) solution: 92 mM NMDG, 2.5 mM KCl, 1.25 mM NaH_2_PO_4_, 30 mM NAHCO_3_, 20 mM Hepes, 25 mM glucose, 2 mM thiourea, 5 mM NA ascorbate, 3 mM NA pyruvate, 0.5 mM CaCl_2_, and 10 mM MgSO_4_·7H_2_O (315 mosmol liter^−1^, pH 7.3 to 7.4). Horizontal brain slices (250 μm thick) were cut and incubated in NMDG solution. Slices were incubated at 34°C for 12 to 15 min in NMDG following cutting and returned to Hepes ACSF at room temperature until recording. The Hepes ACSF solution comprised 92 mM NaCl, 2.5 mM KCl, 1.25 mM NaH_2_PO_4_, 30 mM NaHCO_3_, 20 mM Hepes, 25 mM glucose, 2 mM thiourea, Na ascorbate, Na pyruvate, 2 mM CaCl_2_, and 2 mM MgSO_4_·7H_2_0 (315 mosmol liter^−1^, pH 7.3 to 7.4). The ACSF bath during recording contained 126 mM NaCl, 3 mM KCl, 1.25 mM NaH_2_PO_4_, 2 mM MgSO_4_, 26 mM NaHCO_3_, 10 mM dextrose, and 2 mM CaCl_2_ (315 to 320 mosmol liter^−1^, saturated with 95% O_2_/5% CO_2_). The submersion recording chamber was held at 34°C (TC-324B, Warner Instruments). Electrodes were filled with 135 mM potassium gluconate, 2 mM KCl, 4 mM NaCl, 10 mM Hepes, 0.2 mM EGTA, 4 mM adenosine 5′-triphosphate–Mg, 0.3 mM guanosine 5′-triphosphate–tris, and 10 mM phosphocreatine-tris (295 mosmol liter^−1^, pH 7.25). KOH (1 M) was used to adjust pH of the internal solution. The approximate bath flow rate was 2 ml min^−1^, and the recording chamber held ∼5-ml solution.

The TRN was visualized under ×4 magnification, and single or pairs of adjacent TRN cells with strong ChroME-mRuby expression nearby but without preference to core or shell (*58*) or sensory sector (*100*) were identified and patched under 40× IR-DIC optics (SliceScope, Scientifica, Uckfield, UK). Cell type was determined by mRuby epifluorescence in the patched neurons. Cells with low fluorescent contrast were avoided. The mRuby reporter was excited by a 560-nm diode delivered through the objective (CoolLED pE-300). Voltage signals were amplified and low-pass filtered at 8 kHz (MultiClamp, Axon Instruments, Molecular Devices, Sunnyvale, CA, USA), digitized at 20 kHz with custom MATLAB routines controlling a National Instruments (Austin, TX, USA, USB6221 DAQ board), and data were stored for offline analysis in MATLAB (MathWorks, R2023b, Natick, MA, USA). Recordings were made in whole-cell current-clamp mode. Negative current was used to maintain cells at −70 to −60 mV during recordings. Pipette resistances were 5 to 9 MΩ before bridge balance; recordings were discarded if access resistance exceeded 25 MΩ. Voltages are reported uncorrected for the liquid junction potential.

### Stereotaxic surgery

SOM-Cre (Jax, 013044), PV-Cre (Jax, 008069), or PV-Cre-CRISPR-Cas9-GFP mice were anesthetized via inhaled isoflurane (0.8 to 1.2%) and 0.1 ml of buprenorphine (0.015 mg/ml) via intraparietal injection. Mice were positioned on a heated pad and in a stereotax (Kopf). After exposure of the skull, a dental drill was used to open a hole above the injection site. Then, AAV9-CAG-DIO-ChroME-st-P2A-H2B-mRuby (titer, 2.7 × 10^13^ GC/ml) was injected at a rate of 100 nl/min (WPI UMP-3). pAAV-CAG-DIO-ChroME-ST-P2A-H2B-mRuby3 was a gift from H. Adesnik (Addgene, viral prep no. 108912-AAV9; http://n2t.net/addgene:108912; RRID:Addgene_108912). Viral vectors used for knocking down Cx36 or Cx29 were designed by D. Uygun and R. Basheer as described previously (*99*). Briefly, the PX552 plasmid (Addgene, plasmid no. 60958) was modified to encode triple U6-sgRNA cassettes each targeting a distinct locus of the target genes. The GFP sequence was replaced by the sequence encoding the blue fluorescent protein (BFP). The coordinates for injections were −1.35 AP, ±2.1 ML, and −3.0 and −3.2 DV for TRN and −0.5 AP, ±3.0 ML, and −2.0 and −2.6 DV for somatosensory cortex. We injected a total of 600 nl of virus for TRN and 800 nl of virus for somatosensory cortex. Following surgery and scalp suturing, mice were given an intraperitoneal injection of 0.1 ml of carprofen (0.5 mg/ml) and 1 ml of saline and then monitored for recovery. Mice were given at least 2 weeks for viral expression before experiments. For neonate surgery, P0 mice were anesthetized via hypothermia on a cold metal plate, and the coordinates for injection targeting TRN were −1.4 AP, ±1.9 ML, and −1.4 and −1.9 DV.

### Image processing

All fluorescence images were modified in Fiji and pseudocolored to facilitate visualization. All infrared differential interference contrast images were flat-field corrected.

### Opto-δ*L*

For the rheobase current necessary to measure δ*L*, we used the smallest stimuli that drove spiking at least 80% of the time, with the CV of latency less than 0.2. Trials were excluded if latency was less than 20 ms for rheobase stimuli.

To perform single-cell optogenetic stimulation, we used a 560-nm LED (Mightex) and digital mirror device (Mightex Polygon 1000) to direct light to an area the size of an individual soma (7- to 8-μm diameter circle). The area used covered the entire soma without extending beyond the edge of the soma. Before any experiments, we patched an opsin-expressing cell to determine an intensity of light that was sufficient to drive bursts for a 50 ms stimulus, and we then used a photostimulus that was 50% stronger for all subsequent experiments in that slice. Light intensity ranged from 3.7 to 99.8 mW/mm^2^ with most experiments occurring in 6.2 to 25.0 mW/mm^2^ range.

Single-cell optogenetic stimulation is confounded by off-target excitation by light scatter for both one-photon and two-photon stimulation paradigms (*79*–*81*). For the purposes of mapping synaptic connections, scatter-induced photostimulation of the patched neuron is a potential confound. Depolarization from photostimulation caused by light scatter is directly proportional to the distance between the patched neuron and focal stimulation (fig. S3). Opto-δ*L* is well suited to overcome the problem of unintended photostimulation to the patched neuron because the method quantifies latency of spikes rather than voltage changes. Consequently, comparing latency when a nearby neuron is photostimulated to latency when an equidistant empty control location is photostimulated allowed us to distinguish the contribution of GJs to spike times.

After patching TRN neurons, we used one of the two methods to make measurements that would control for the effects of light scattering and then to compute δ*L*:

1) Direct corrections to δ*L*: We delivered a rheobase stimulus to the patched neuron alone 10 times. Interleaved between those stimulations, we gave the neuron a rheobase stimulus while also focally photostimulating either a nearby mRuby^+^ neuron or an equidistant empty spot. Offline, we measured the latency of spikes from stimulation onset for each condition. We report δ*L* as the change in latency induced by focal photostimulation of the nearby neuron minus the change in latency induced by photostimulation of an empty area.

2) Correction to *I*_app_: We applied focal photostimulation ten times to an empty location equidistant from the patched neuron as the nearby neuron of interest. We measured the average depolarizing current that resulted from the light scattering. We then adjusted rheobase *I*_app_ by average current caused by light scatter (fig. S4) and used that adjusted *I*_app_ when photostimulating the nearby mRuby^+^ or an equidistant empty location. Offline, we calculated δ*L* by comparing latency changes between when light targeted the empty control location ($\bar{t_{1}}$) compared with light targeting the mRuby^+^ neuron ($\bar{t_{2}}$).

We directly corrected δ*L* for 27 homocellular network measurements, and we used correction to *I*_app_ for 51 homocellular network measurements.

### Numerical analysis and statistics

To calculate δ*L*, we used the average spike latency from control trials ($\bar{t_{1}}$) and average spike latency when a nearby neuron was photostimulated ($\bar{t_{2}}$)

$$\delta L=\frac{100*\left(\bar{t_{1}}-\bar{t_{2}}\right)}{\bar{t_{1}}}$$

We used Friedman’s tests on the sets of latencies resulting from stimulation with rheobase only, rheobase with blank spot photostimulation, and rheobase with neighbor photostimulation for homocellular networks that used direct correction by blank spot stimulation, and we used Wilcoxon signed-rank tests for post hoc pairwise comparisons of spike latencies. We used Wilcoxon signed-rank tests to compare latencies in experiments corrected by *I*_app_. Pearson’s correlation coefficient was used to test correlations. We used Mann-Whitney *U* tests for comparisons between neuron types, and we used Fisher’s exact tests to compare coupling rate and electrical synapses counts per network. For multiple comparisons, we used Holm-Bonferroni correction and report the corrected *P* value. Statistical tests were considered significant when *P* < 0.05.

### Networks

In our analysis of network size, we required three or more connections tested from a hub cell to be considered as a network.
